# A Plea for Medical Treatment in Certain Surgical Cases

**Published:** 1909-10-02

**Authors:** Dyce Duckworth


### A PLEA FOR MEDICAL TREATMENT IN CERTAIN SURGICAL CASES.

By SIR DYCE DUCKWORTH, BT., M.D., LL.D.

While fully appreciating the remarkable advance and improvements in modern surgery, I venture to think that there may be observed a tendency on the part of some surgeons to dispense with any aid that may be afforded by medicinal and other remedial measures in the treatment of patients that have been submitted to operation.

There may be several reasons for this. One is probably due to the fact that some cases ultimately come under surgical care which have previously been medically treated without benefit, sometimes, perhaps, for an undue length of time. An operation at once secures relief, and the subsequent dressings are supposed to comprise all that is necessary to secure perfect recovery. Another reason, which certainly obtains in some instances, is that operating surgeons have now a smaller experience, and therefore less confidence, in the value of medicines than the older surgeons had. I think that these facts have to be recognised, and I am therefore concerned to put in a plea for requisite and appropriate measures of therapeutics in certain cases after surgical operations. To illustrate my contention, I will mention the circumstances of a case which has come under my notice. A young man suffering from an empyema is committed to a surgeon for treatment. The usual operation is performed with great relief to the patient. A free discharge of pus continues for some weeks, and several dressings are required each day. In the meantime the patient is kept on a low diet and receives no medicine. This is, indeed, *pure* surgery.

In the presence of free depuration, a physician always bethinks himself of the necessity of a very nutritious dietary, including, not seldom, some alcoholic support, such as porter; and is well aware of the great benefit to be gained by employing five or eight grains of quinine with dilute sulphuric or phosphoric acid daily. Under this treatment the discharge of pus rapidly lessens, and sound recovery ensues. Without it the patient's recovery is retarded, and wasting and enfeeblement ensue. These measures are imperative, especially if the benefit of a seaside convalescent home cannot be promptly secured.

The modern surgeon sees little or nothing of depuration as a sequel of his operations, and when these are carried out on persons otherwise in sound health there may be no need for special medication. In strumous subjects who may be benefited by operative measures the case is very different. I need hardly state that there is no universal rule in this matter. The patient and his personal peculiarities always constitute a problem for consideration in each instance.

I am inclined to fear that some of our younger brethren, who are specially captivated by the promptness and certainty of modern surgical procedure, are beginning to believe that nothing beyond this is necessary for the restoration to sound health of the ordinary surgical patient, and are thus failing to acquire experience in the use of other therapeutical measures which are certainly needed in many cases. I maintain that the skill of the surgeon has, not seldom, to be supported and reinforced by the art of medicine, and for a period long after the operator has retired from the patient. This is not sufficiently recognised at the present time, and it is necessary to declare that if drugs and particular dietaries are of small avail in cases which plainly demand surgical interference for their relief, that fact constitutes no reason for disregarding the benefits to be derived from them at a subsequent stage of their progress.

Lastly, I would add that the unwise teaching of the present day in regard to the employment of alcoholic beverages is, in my belief, harmful in the best interest of sound convalescence from many ailments. This is a matter for the sick of purely clinical skill, demanding as much care and attention, given with an open mind, as the prescription of any other remedial measures, and it may not be ignored in deference to any prevalent wave of public opinion.

